# Protocol to study DNA strand breaks during development and apoptosis using *in situ* nick translation in *Drosophila*

**DOI:** 10.1016/j.xpro.2025.103921

**Published:** 2025-06-26

**Authors:** Deepak Maurya, Bama Charan Mondal

**Affiliations:** 1 Cytogenetics Laboratory, Department of Zoology, Institute of Science, Banaras Hindu University, Varanasi 221005, India

**Keywords:** Cell Biology, Developmental biology, Microscopy, Model Organisms

## Abstract

Cellular stress causes DNA strand breaks that are typically repaired to maintain homeostasis and regulate cell fate. However, unrepaired DNA breaks can be lethal, leading to cell death. Here, we present a protocol to study DNA strand breaks in *Drosophila* during development and apoptosis using *in situ* nick translation. We describe the steps for labeling DNA strand breaks using digoxigenin (DIG)-labeled nucleotide (DIG-11-dUTP) and visualizing them with anti-DIG immunostaining. We then detail procedures for mounting, imaging, and analysis.

For complete details on the use and execution of this protocol, please refer to Maurya et al.^1^ and Rigby et al.^2^

## Highlights

• Step-by-step guide for preparing samples and slides for *in situ* nick translation assay
• Instructions for measuring the extent of DNA strand breaks in particular tissues
• Steps for detecting cell death by combining *in situ* nick translation with immunostaining

## Abstract (editor highlights)

Publisher’s note: Undertaking any experimental protocol requires adherence to local institutional guidelines for laboratory safety and ethics.

## Graphical abstract

**Figure:**
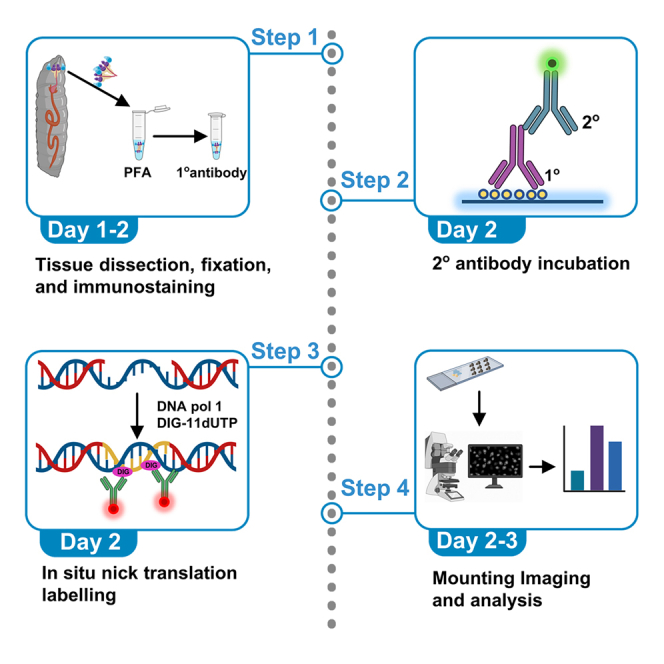

## Before you begin

In situ nick translation (ISNT) is a highly sensitive technique for detecting DNA strand breaks. In nick translation, the DNA break site is synthesized to a 3′-hydroxyl end in the presence of template using DNA polymerase I. During this process, the labeled nucleotide is incorporated into the synthesizing strand, and detection of this labeled nucleotide confirms the DNA strand breaks. It has been utilized for various applications, including making probes for the hybridization technique and molecular cytogenetics, as well as detecting apoptosis and necrosis mediated cell death.^2^^,^^3^^,^^4^^,^^5^^,^^6^^,^^7^ This protocol can easily detect both apoptotic and non-apoptotic DNA strand breaks. Here, we have standardized to label DNA strand breaks in the *Drosophila* larval hematopoietic organ called the lymph gland, and efficiently detected them during development. Additionally, we observed nick translation in eye imaginal discs during cell death. This low-cost, kit-free protocol can also be applied to other *Drosophila* tissues.

We have detailed the complete protocol here, including solution preparation, dissection of the *Drosophila* larval tissues, immunostaining, incubation and labeling, imaging, and data analysis.

### Institutional permission

This study did not require institutional approvals for the *Drosophila* model system, but researchers should obtain necessary approvals as per their institutional guidelines. This protocol uses the transgenic *Drosophila melanogaster* (fruit fly) maintained under specific, pathogen-free, and well-maintained laboratory conditions approved by the Institutional Biosafety Committee at Banaras Hindu University. The protocol used samples from both male and female flies without distinction.

### Cross setup

**Timing: 5 days**

1. Set up the experiment and control crosses synchronously to ensure sufficient larvae of desired genotype come out; however, avoid overcrowding.
2. Crosses that involve the Gal4/UAS system placed in a BOD (biological oxygen demand) incubator (PHCBI Model #MIR-554-PE) set to 29°C.

### Solution preparation (for recipe, check the materials and equipment section)

**Timing: 2 days**

3. The following solutions can be kept as stock solutions and need to be prepared in advance:
  a. 1× PBS.
  b. 1× PBS with 0.3% Triton X-100 (PBST).
  c. Stock solution of DAPI (1 mg/mL).
  d. Make the stock solution of dNTPs.
  e. Dissolve rhodamine-conjugated anti-DIG antibody (100 μg/mL).
  f. Blocking solution.
  g. DABCO solution.
4. The following solutions must be prepared freshly. These solutions should not be stored:
  a. 4% paraformaldehyde fixative solution.
  b. 1× PBS with magnesium chloride.
5. The following solutions must be prepared immediately:
  a. Primary antibody solution.
  b. Secondary antibody solution (anti-rabbit AF647).
  c. Nick-translation reaction mixture.
  d. Rhodamine-conjugated anti-Digoxigenin antibody working solution.

## Key resources table

**Table:** 

REAGENT or RESOURCE	SOURCE	IDENTIFIER
**Antibodies**		

Anti-Digoxigenin-Rhodamine, Fab fragments (dilution 1:100)	Sigma	Cat# 11207750910
Goat-rabbit Alexa Fluor 647 (dilution 1:100)	Invitrogen	Cat# A32733; RRID:AB_2866492
Rabbit-Histone H2AvD phosphoS137 antibody (dilution 1:100)	Rockland	Cat# 600-401-914; RRID:AB_828383

**Chemicals, peptides, and recombinant proteins**		

DAPI (4′,6-diamidino-2-phenylindole, dihydrochloride)	Invitrogen	Cat# D1306
4% paraformaldehyde (PFA)	Thermo Fisher Scientific	Cat# 28908
Digoxigenin-11-dUTP, alkali-labile	Sigma	Cat# 11573152910
Deoxynucleotide set, 100 mM	Sigma	Cat# DNTP100-1KT
DABCO (1,4-diazabicyclo [2.2.2]octane)	Sigma	Cat# D27802
DNA polymerase I	New England Biolabs	Cat# M0209S
Fetal bovine serum	HiMedia	Cat# RM9955
Triton X-100	Sigma	Cat# T8787
Bovine serum albumin	SRL	Cat# 83803
Thiomersal	SRL	Cat# 85090
Magnesium chloride	SRL	Cat# 91417
Glycerol	SRL	Cat# 42595

**Experimental models: Organisms/strains**		

*D. melanogaster*: *w*^*1118*^ (3–5 days old adult, mixed sexes)	Bloomington Drosophila Stock Center	RRID: BDSC_5905
*D. melanogaster*: *e33c-Gal4* (3–5 days old adult, mixed sexes)	Maneesha Inamdar lab	N/A
*D. melanogaster*: *GMR-hid* (3–5 days old adult, mixed sexes)	Cytogenetics Lab	Grether et al.^8^
*D. melanogaster*: *GMR-Gal4* (3–5 days old adult, mixed sexes)	Cytogenetics Lab (Freeman^9^)	RRID: BDSC_1104
*D. melanogaster*: *UAS-127Q* (3–5 days old adult, mixed sexes)	Cytogenetics Lab (Gift from P. Kazemi-Esfarjani)	Kazemi-Esfarjani and Benzer^10^

**Software and algorithms**		

ImageJ	NIH	https://imagej.net/ij/
Prism 9	GraphPad	https://www.graphpad.com/scientific-software/prism/
Zen Software Version 3.4	Zeiss	https://www.zeiss.com/microscopy/us/products/software/zeiss-zen.html
Adobe Photoshop 2021	Adobe	version 22.4.2
Adobe Illustrator cc 2018	Adobe	version 22.1
Microsoft Word, Excel, PowerPoint	Microsoft 2019	Microsoft 2019

**Others**		

Microscope glass slides	Mowell	Cat# B098K8PSDY
Cover glass (22 × 22 mm)	Blue star	N/A
Dissecting tweezers	Dumont	Catt# 72701-12
Micropipette p1000, p200, and p20	Gilson	Cat# FA10006M, FA10005M, FA10003M, FA10001M
Disposable tips (1,000 μL, 200 μL, and 20 μL)	Tarson	Cat# 521020, 521010, and 521000
Centrifuge tubes (15 mL and 50 mL)	Tarson	Cat# 546021 and 546043
Thermocycler	Thermo Fisher Scientific	Cat# 4375786
*Drosophila* incubator	PHCBI	Cat# MIR-554-PE
Microcentrifuge tube (1.5 and 0.2 mL)	Tarson	Cat# 500010 and 510052
Stereomicroscope	Zeiss	Cat# Stemi 508
Confocal microscope	Zeiss	Cat# LSM-900
Fine point paintbrushes	Camel	Cat# 0030
Nail polish	Candy	Cat# 1562/COS

## Materials and equipment

**Table:** 1× Phosphate Buffered Saline (PBS)

Reagent	Final concentration
NaCl	137 mM
KCl	2.7 mM
Na_2_HPO_4_	4.3 mM
KH_2_PO_4_	1.5 mM

Note: Store at 4°C up to a month; pH:7.4. See troubleshooting 1.

**Table:** 4% Fixative solution

Reagent	Final concentration	Amount
16% Paraformaldehyde	4% Paraformaldehyde	250 μL
1× PBS	N/A	750 μL
**Total**	**N/A**	**1,000 μL**

Note: Dilute before use, and store at 4°C up to a week. Paraformaldehyde is light sensitive so store in amber vials. See troubleshooting 1 and 3.

**Table:** 1× PBS-Triton X-100

Reagent	Final concentration	Amount
1× PBS	N/A	100 mL
Triton X-100	0.3%	0.3 mL

Note: Store at 4°C up to a month. See troubleshooting 2.

**Table:** 1× PBS with Magnesium chloride

Reagent	Final concentration	Amount
1× PBS	N/A	100 mL
Magnesium chloride	0.5 mM	0.0101 g

Note: Make it fresh; it can be stored up to a month at 4°C.

**Table:** Nick-translation reaction mixture

Reagent	Final concentration	Stock	Amount
dATP	50 μM	1 mM	1.25 μL
dGTP	50 μM	1 mM	1.25 μL
dCTP	50 μM	1 mM	1.25 μL
dTTP	35 μM	1 mM	0.875 μL
DIG-dUTP	15 μM	100 μM	3.75 μL
DNA Polymerase I Buffer	1×	10×	2.5 μL
DNA Polymerase I	40 U/mL	10,000 U/mL	0.1 μL
Water	N/A	N/A	14.025 μL
**Total**	**N/A**	**N/A**	**25 μL**

Note: Make just before incubation of the sample and do not store. See troubleshooting 2 and 3.

**Table:** Blocking solution

Reagent	Final concentration	Amount
Fetal Bovine Serum	10%	10 mL
Bovine Serum Albumin	0.1%	0.1 g
Thiomersal	0.02%	0.02 g
Triton X-100	0.1%	0.1 mL
Deoxycholic Acid	0.1%	0.1 g
1× PBS	N/A	90 mL
**Total**	**N/A**	**100 mL**

Note: Store at −20°C up to a month in small aliquots and avoid freeze-thaw. See troubleshooting 1.

**Table:** Primary antibody solution (anti-γH2Av)

Reagent	Final concentration	Amount
Rabbit-Histone H2AvD phosphoS137 Antibody (1.1 mg/mL)	1:100 dilution	1 μL
Blocking solution	N/A	99 μL
**Total**	**N/A**	**100 μL**

Note: Make fresh just before use and do not store.

**Table:** Secondary antibody solution (anti-rabbit AF647)

Reagent	Final concentration	Amount
Goat-rabbit Alexa Fluor 647 (2 mg/mL)	1:200 dilution	0.5 μL
Blocking solution	N/A	99.50 μL
**Total**	**N/A**	**100 μL**

Note: Make fresh just before use and do not store. The fluorophore is light sensitive, so make it in amber microcentrifuge tubes.

**Table:** Rhodamine-conjugated anti-DIG antibody working solution

Reagent	Final concentration	Amount
Rhodamine (100 μg/mL)	1:200 dilution	1 μL
Blocking solution	N/A	199 μL
**Total**	**N/A**	**200 μL**

Not**e:** Make fresh just before use and do not store. The fluorophore is light sensitive, so make it in amber microcentrifuge tubes. See troubleshooting 2 and 3.

**Table:** DABCO solution

Reagent	Final concentration	Amount
DABCO	2.5%	0.25 g
Glycerol	75%	7.5 mL
Distilled water	N/A	2.5 mL
**Total**	**N/A**	**10 mL**

Note: Store at −20°C up to a month.

***Alternatives:*** We provide a list of suppliers for standard molecular biological buffers, reagents, and equipment. Similar products from other suppliers can usually be substituted without any issues.

## Step-by-step method details

### Dissection, fixation, and permeabilization

**Timing: 2–3 h**

**Figure 1:**
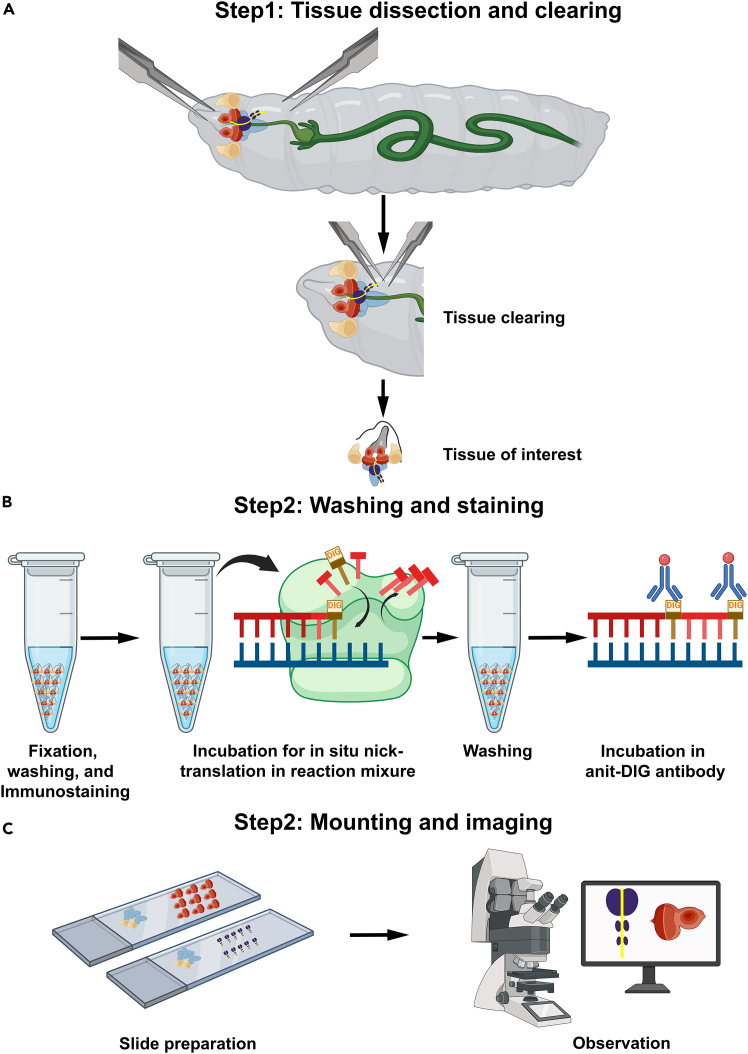
Schematic representation of the step-by-step method details (A) Dissection of desired larval tissue in 1× PBS and removal of the debris. (B) Tissues were fixed in 4% PFA, immunostaining performed, and washed with 0.3% PBST thrice. Tissues were incubated in the nick-translation reaction mixture for 2 h at 37°C and washed thrice with 0.3% PBST. After incubation for 2 h in blocking solution, tissues were incubated in rhodamine-conjugated anti-DIG antibody solution containing DAPI for 2 h at 25°C. (C) After washing thrice with PBST, the lymph glands or eye discs were separated from the rest of the tissues, then mounted on the slide and observed under the confocal microscope.

This section is very crucial, which describes the isolation of desire tissues, fixation, and permeabilization (Figure 1).

1. Dissection.
  a. Dissect and remove the brain complex using tweezers. It contains the eye-antennal disc, central nervous system, lymph gland, and a few other associated tissues (Figure 1).^11^^,^^12^
  b. Transfer the dissected tissues to a clean microcentrifuge tube (200 μL) containing 1× phosphate-buffered saline (PBS), and place on ice.
  c. Dissection is performed for no more than 20 min in chilled 1× PBS at room temperature (RT) (24°C–25°C).

***Note:*** Cold PBS provides extra time for dissection; however, prolonged dissection may cause tissue deterioration. See troubleshooting 1.

2. Fix 10–20 brain complexes in freshly prepared 4% paraformaldehyde (PFA) solution in 1× PBS for 20 min at RT.

***Note:*** It is a critical step, and in longer fixation, tissues may become brittle, leading to improper or no staining. See troubleshooting 1, 2, and 4.

3. Wash tissues with 0.3% PBST (1× PBS + 0.3% Triton X-100) three times for 10–15 min each.

***Note:*** Washing using the Triton X-100 causes permeabilization; less washing may not allow penetration of enzymes/antibodies within tissues or cells. See troubleshooting 2.

### Primary antibody incubation

**Timing: 15–17 h**

This section describes the steps for primary antibody incubation for co-staining with nick translation.

4. Incubate the tissues for 2 h in blocking buffer at RT.

***Note:*** Incubation in blocking solution before antibody incubation reduces the background signal and increases the signal-to-background ratio. See troubleshooting 1.

5. Remove the blocking solution and add 50 μL of primary antibody (anti-γH2Av antibody) solution diluted in blocking for 12 h at 4°C.

***Note:*** 12 h incubation at 4°C gives better results; alternatively, 1-h incubation in a 37°C incubator can be performed.

### Secondary antibody incubation

**Timing: 4–5 h**

This section describes the steps for secondary antibody incubation for co-staining with nick translation.

***Note:*** All steps must be performed in the dark condition.

6. Remove the primary antibody and wash tissues thrice with 0.3% PBST for 10–15 min each at RT.

***Note:*** This step is crucial to remove the non-specific signal.

7. Incubate the tissues in blocking solution for 1 h at RT.
8. Remove the blocking solution and incubate in 50 μL of secondary antibody (anti-rabbit AF647) diluted in blocking solution for 2 h at RT.

***Note:*** Select a secondary antibody that does not interfere with the nick labeling signal. In this case, we utilized anti-rabbit AF647 to identify DNA damage repair and anti-DIG Rhodamine to visualize nick labeling, ensuring that the excitation wavelengths for both signals do not overlap. See troubleshooting 2 and 3.

9. Remove the secondary antibody solution and wash tissues thrice with 0.3% PBST for 10–15 min each at RT.

### Incubation in the reaction mixture

**Timing: 4–5 h**

This is very crucial section that describes the steps related to synthesis of DNA strand at the break sites with labeled nucleotide (Figure 1).

***Note:*** All steps must be performed in the dark condition.

10. Wash tissues twice with 1× PBS supplemented with 0.5 mM magnesium chloride for 5 min each.

***Note:*** Magnesium ion enhances the efficiency of the enzyme (DNA polymerase I).

11. Prepare the nick translation reaction mixture as describe is materials and equipment section.

***Note:*** Prepare the reaction mixture just before incubation on ice. See troubleshooting 2 and 3.

12. Add a 25 μL reaction mixture in each 200 μL microcentrifuge tube containing the tissues.
13. Transfer the microcentrifuge tube to the thermocycler and run the program for 2 h at 37°C. Alternatively, the microcentrifuge tube can be incubated in a water bath for 2 h.

***Note:*** A water bath may not be as effective as a thermocycler. See troubleshooting 1, 2, 3, and 4.

14. After incubation, remove the reaction mixture and wash with 0.3% PBST, three times for 10 min each.
15. Incubated tissues for 1 h in blocking solution at RT.

### Labeling for nick translation and DAPI staining

**Timing: 2.5–3 h**

This section describes the labeling of anti-DIG antibody with DIG labeled nucleotide (Figure 1).

***Note:*** All steps must be performed in the dark condition.

16. Incubate the tissues for 2 h at RT with the rhodamine-conjugated anti-DIG antibody (Anti-Digoxigenin-Rhodamine, Fab fragments, 0.5 μg/mL diluted in blocking solution) containing the DAPI (1 μg/mL). See troubleshooting 2, 3, 4, and 6.
17. After incubation, remove the antibody solution and wash with 0.3% PBST, three times for 10 min each. See troubleshooting 2 and 3.

#### Mounting

**Timing: 15–20 min**

This section of protocol provides the instruction about the preparation of slide after the staining of tissues and storage of prepared slides (Figure 1).

***Note:*** All the steps in this protocol should be performed in low light condition that minimizes the photo-bleaching of fluorescent signals.

18. Transfer the tissues to a clean glass slide and remove the excess PBST (Figure 1).
19. Immediately add a drop of mounting media (DABCO) on the tissues.
20. Detach the lymph gland and eye disc from the brain complex, and correctly arrange them on the slide.

***Note:*** Final removal of tissues in PBST may degrade tissues as they can dry out quickly. Therefore, using mounting media prevents tissue degradation while mounting.

21. Apply the coverslip gently and seal the edges of the coverslip with nail polish.

***Note:*** Proper sealing of the coverslip is important to prevent leakage of mounting media. See troubleshooting 2.

22. Store the mounted slide in a slide holder, scan it under the confocal microscope, or store it at −20°C.

***Note:*** Let the nail paint dry before observation; otherwise, coverslips may be removed/slid, or stuck to the microscope objectives.

### Imaging and analysis

**Timing: 2–3 h**

The section provides the detail steps for the acquisition of images of mounted slides using confocal microscope, also discuss the imaging and analysis of acquired images.

23. Acquire Images of the prepared slide under the confocal microscope using the required laser channels.

***Note:*** Anti-Digoxigenin-Rhodamine (Sigma Cat# 11207750910) used in this study, which excites at the red channel (λ545), and anti-rabbit AF647 excitation at far-red channel (λ647). See troubleshooting 6.

24. Set the pinhole on 1, and optimize the gain to avoid excessive or weak signals. Select the detection range for the photomultiplier tube (PMT) detectors with respect to individual laser channel.

***Note:*** Setting of detection range for detectors are important that avoid the detection of overlapping signals of the other channels. The interval selection for Z section should be appropriate so that the nuclei can be detected in optical sections. Here, we have used 2 microns interval so that the nuclei can be detected in more than one optical sections. See troubleshooting 6.

25. The Z section was selected based on tissue thickness, and the optical section interval was set to 2 microns.
26. Save all the images in appropriate formats depending on the confocal microscope.
27. Open images using ImageJ software and quantify the nick-positive cells (also see quantification and statistical analysis section).
28. Perform statistical analysis using GraphPad Prism or MS Excel and plot a graph (also see quantification and statistical analysis section).

## Expected outcomes

**Figure 2:**
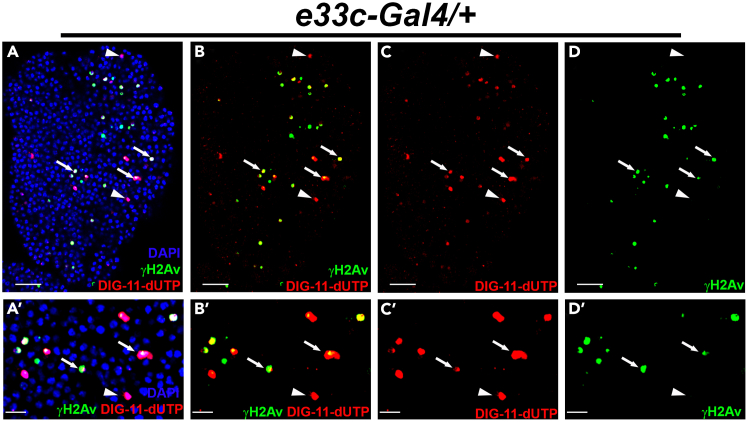
In situ nick translation also labels the dying cells (A–D′) Nick positive cells (red) co-localized with γH2Av positive cells (green) in control lymph gland (*e33c-Gal4/+*) with nuclear DAPI staining (blue) (A and A′), without DAPI (B and B′), only nick translation (C and C′) and only γH2Av staining (D and D′). The lymph gland was dissected from wandering third instar larvae, and all images shown are single optical sections. The arrow indicates the co-localization of γH2Av staining and nick translation, and the arrowhead shows only nick translation. The scale bar represents 25 μm for the complete lymph gland lobe and 10 μm for all cropped high magnification images.

In situ nick translation (ISNT) is a classical and powerful technique for labeling DNA strand breaks, where the break sites are synthesized using *E. coli* DNA polymerase I.^2^^,^^5^ During synthesis, DNA polymerase I adds a labeled nucleotide to the synthesizing DNA strand. The nucleotide can be labeled with radioactive or fluorescent material and further detected by autoradiography or fluorescent/confocal microscopy.^1^^,^^4^^,^^13^^,^^14^^,^^15^ Developmental DNA strand breaks if repaired on time that can regulate cell differentiation.^1^^,^^14^ However, if damaged DNA is not repaired on time, it can lead to cell death.^16^ We have also observed during the terminal differentiation of *Drosophila* lymph gland progenitors, caspase-mediated DNA strand breaks occur that lead to DNA damage response (DDR) and promotes macrophage-type cell differentiation.^1^ If they remain unrepaired, cells die, as marked by strong TUNEL-positive cells. ISNT labeling and DDR marker γH2Av immunostaining in the *Drosophila* lymph gland (*e33c-Gal4/+*) reveal that nick translation labels the cells having high DDR; however, strongly labels ISNT in the cells where DDR is absent (Figure 2). These non-DDR with strong ISNT positive cells resemble the TUNEL-positive cells in the lymph gland.^1^ Thus, ISNT can be used as an alternative method of TUNEL to detect the dying cells. Here, we have also standardized the ISNT protocol using *Drosophila* eye-antennal imaginal discs. We have utilized DIG-labeled dUTP (DIG-dUTP), which is detected by a fluorescently labeled (rhodamine) anti-DIG antibody, and the discs’ nuclei are stained with DAPI.

**Figure 3:**
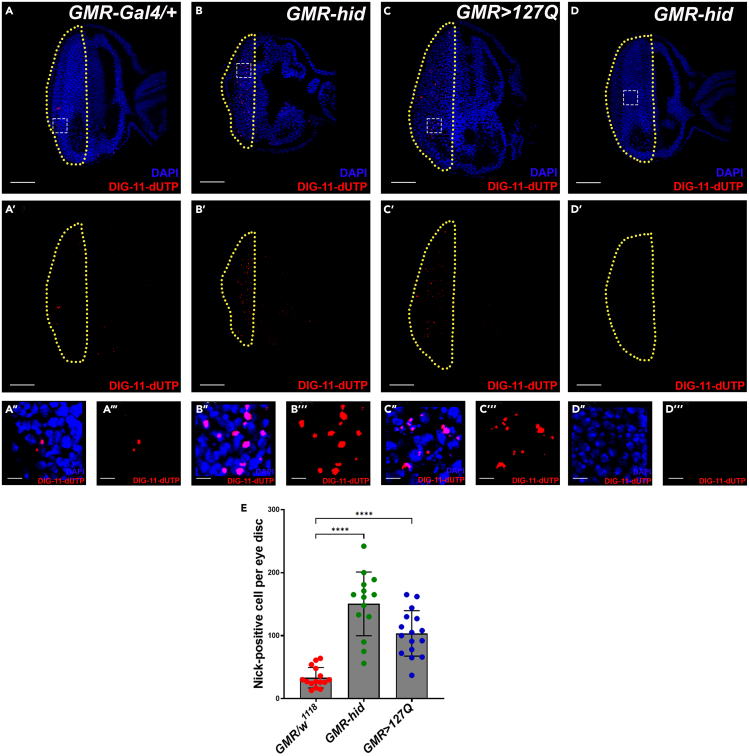
Numbers of nick-positive nuclei increase in the eye disc during cell death (A–A‴) A few nick-positive nuclei (red) were found in the control (*GMR-Gal4/+*) (n=15) eye disc (A). Also, shown in the red channel only (A′), and in high magnification (A″ and A‴). (B–B‴) Nick-positive nuclei (red) increase significantly upon apoptotic induction (*GMR-hid*) (n=14) (B). Also, shown in the red channel only (B′), and in high magnification (B″ and B‴). (C) Neurodegeneration also causes increased nick-positive nuclei (red) (*GMR>127Q*) (n=16) (C). Also, shown in the red channel only (C′), and in high magnification (C″ and C‴). (D) Absence of DNA polymerase I in the reaction mixture does not label any dying nuclei (*GMR-hid*), and it serves as a negative control of the experiment (D). Also, shown in the red channel only (D′), and in high magnification (D″ and D‴). (E) Quantification of nick-positive nuclei per eye disc (A–C‴). All eye discs dissected out from wandering third instar larvae, and all images shown are single optical sections. Nick translation marked in red and nuclei stained with DAPI (blue). The yellow line demarcated the GMR-positive area of the eye disc, and the white dotted square marks the region of the disc shown in high magnification. The scale bar represents 50 μm for all the eye disc images and 5 μm for all cropped high magnification images. ∗∗∗∗P < 0.0001 Error bars, mean ± SD. All images represent 3 or more independent biological experiments, and ‘n’ represents the number of lymph gland lobes.

Programmed cell death (apoptosis) occurs during the development of the *Drosophila* eye disc and can be enhanced by the overactivation of cell death machinery.^17^^,^^18^^,^^19^ DNA fragmentation is one of the signs of apoptosis.^20^^,^^21^^,^^22^ We have labeled the developmental DNA breakage in the control eye disc (*GMR-Gal4/+*),^9^ using the ISNT protocol (Figures 3A–3A‴ and 3E). Overexpression of the proapoptotic gene *hid* in the eye disc using the *GMR* promoter (*GMR-hid*)^8^ causes severe induction of apoptotic pathway and leads to cell death, as evidenced by the significant increase in the number of nick-positive nuclei compared to the control (*GMR-Gal4/+*) (Figures 3A–3B‴ and 3E). We have further validated the staining protocol for neurodegenerative disease conditions, where cell death is one of the indicators of neurodegeneration.^23^^,^^24^^,^^25^^,^^26^ We expressed polyglutamine repeats (CAG) in photoreceptors of the eye disc (*GMR-Gal4;UAS-HTT.127Q*), which leads to a significant increase in cell death.^10^ Therefore, we found significantly high nick-positive nuclei in the *GMR>HTT.127Q* eye disc compared to the control (*GMR-Gal4/+*) (Figures 3A–3A‴, 3C–3C‴, and 3E). We also confirmed the nick translation protocol using a negative control, where the DNA polymerase I is absent in the reaction mixture, and we did not find any staining in the eye disc, even though the disc expresses pro-apoptotic gene (*GMR-hid*) (Figures 3D–3D″).

## Quantification and statistical analysis

Rhodamine fluorescence-positive nuclei marked the DNA damage in the lymph gland and eye disc. For their quantification, the czi format images from the confocal microscope were opened in the Fiji/ImageJ software (NIH, USA) (available at imagej.nih.gov/ij), and the region of interest (front region of morphogenetic furrow of eye disc) was selected for the quantification. The split channel function separated the red (rhodamine) and blue (DAPI) channels. Damaged nuclei showed highly intense rhodamine fluorophore nick labeling and were counted manually using a multipoint tool throughout the Z-stack.

All samples were imaged in a Zeiss LSM 900 laser scanning confocal microscope using Zen Black (version 3.4) software under a Plan Apochromat 40×/1.3 oil objective lens, a zoom of 0.5×, and using a 2.0-micron optical section interval in all images. Adobe Illustrator cc 2018 (version 22.1) and pictures from BioRender were used for schematic model preparation. One representative image is displayed from each experiment, which has been conducted at least three times. With “n” for the number of eye discs or lymph gland primary lobes, the quantifications displayed apply to all the sets examined. GraphPad Prism 9 and Microsoft Excel 2019 were used for all statistical tests for the corresponding experiments. The p-values represent unpaired two-tailed Student’s t-tests to determine statistical significance. The significance level is indicated by an ∗ for p ≤ 0.05, ∗∗ for p ≤ 0.01, ∗∗∗ for p ≤ 0.001, ∗∗∗∗ for p ≤ 0.0001, and by ns for not significant, p > 0.05.

## Limitations

In situ nick translation is a highly sensitive method to label the DNA strand breaks. However, it doesn’t differentiate between the modes of DNA breaks. It can label other sources of DNA breaks, like replication errors, radiation, and chemical treatment. Small DNA break and their repair occur every time in cells, and due to sensitivity, that can also be labeled as background; however, the dying cells will have intense labeling that can be easily differentiated.

## Troubleshooting

### Problem 1

Tissue deterioration (related to steps 1, 2, 4, & 13).

Improper fixation, longer dissection period, pH imbalance in buffers, contamination in blocking solutions, or condensation during the incubation period may lead to tissue deterioration or degradation.

### Potential solution

• Make fresh 4% PFA and ensure all tissues are dipped in the fixative.
• Dissection should be quick and in chilled PBS.
• Make sure that the pH of all buffers is maintained at 7.4.
• Ensure that blocking solutions are not contaminated with bacterial growth and are stored at −20°C.
• Ensure the thermocycler lid is heated, and if the water bath is used, add 30 μL of mineral oil.

### Problem 2

No in situ nick translation labeling or weak fluorescent signal (related to steps 3, 11, 13, & 22).

This problem may arise due to a lower DIG-dUTP or anti-DIG antibody concentration, less tissue permeabilization, improper incubation temperature, or a delay in the observation.

### Potential solution

• Optimize the labeling concentration of dNTPs and anti-DIG antibody. Also check if the stock solution of dNTPs is old, change it with fresh solutions.
• Triton X-100 is viscous, so please ensure no pipetting errors while making PBST. This can increase the number of washes after fixation.
• Strictly maintain the temperature of the water bath, and increase the time for incubation.
• Observe the sample and record images as easily as possible; otherwise, you can store slides at −20°C, but do not keep them for a long time, because the signal may fade.

### Problem 3

High background of ISNT labeling (related to steps 2, 11, & 13). This problem arises due to a high concentration of labeling solution and antibody, a long incubation time, improper washing, or over-fixation of tissues.

### Potential solution

• If the problem remains unresolved, check the labeling solution or antibody and dilute the concentration. Then, increase the number of washes after the incubation.
• Optimize the time of incubation. May increase washing time after secondary antibody incubation.
• Over-fixation causes excess crosslinking and creates small pockets of proteins where antibodies may get stuck. Ensure the timing of fixation and the concentration of PFA. Make a fresh fixation, and do not use an old fixative.

### Problem 4

Variable labeling among tissues (related to steps 2, 13, & 16).

This problem may arise when tissues are not dipped adequately in solutions such as fixative, reaction mixture, or antibody solution.

### Potential solution

• Tissues may float over the solution; therefore, fat bodies may be removed as much as possible.
• Tissues may stick to the inner wall of the tube, which can be removed and dipped into the solutions with a needle or forceps.

### Problem 5

Can’t sure between signal and background.

This problem arises when the signal-to-noise ratio is low.

### Potential solution

• Always use a sample with DNA-damaged nuclei as a negative control in which DNA polymerase I is absent during incubation.
• A positive control is necessary. Use a tissue where DNA damage has already been reported. This sample should have nick-positive nuclei to ensure no issues with the reagents and procedure.

### Problem 6

Overlapping signals fluorophores (related to steps 16, 23, & 24).

This problem arises when fluorophores selected for nick labeling and protein immunostaining have close excitation which causes excitation of the both fluorophore whit same laser. Also, if range for detectors to detect emission light is not accurately set then detector can detect the emission light of another laser channel.

### Potential solution

• The excitation of selected fluorophores that label the ISNT and protein immunostaining should not overlap. For example, if anti-DIG rhodamine is used, then the secondary antibody for protein labeling should not be labeled with fluorophores overlapping with rhodamine excitation and emission spectra.
• Avoid the simultaneous scanning and perform sequential scanning during confocal microscopy, special when fluorophores have very close excitation range.

## Resource availability

### Lead contact

Further information and requests for resources and reagents should be directed to and will be fulfilled by the lead contact, Bama Charan Mondal (bamacharan@bhu.ac.in).

### Technical contact

Technical questions on executing this protocol should be directed to and will be answered by the technical contact, Deepak Maurya (deepakm1295@gmail.com).

### Materials availability

This protocol does not report any newly generated material.

### Data and code availability

The protocol includes all datasets generated or analyzed during this study. Relevant data are available in the study by Maurya et al.^1^ (https://doi.org/10.1016/j.celrep.2024.114251). This paper does not report any original code.

## Acknowledgments

We thank the Cytogenetics laboratory members for sharing reagents and equipment. This study was funded by the 10.13039/501100009053DBT/Wellcome Trust India Alliance Intermediate Fellowship (IA/I/20/1/504931; https://www.indiaalliance.org/), the DBT-Ramalingaswami Fellowship (BT/RLF/Re-entry/08/2016; https://dbtindia.gov.in/), and the Institute of Eminence Scheme, BHU, to B.C.M. and a CSIR fellowship to D.M.

## Author contributions

Conceptualization, D.M. and B.C.M.; methodology, D.M.; investigation, D.M.; formal analysis, D.M.; writing – original draft, D.M.; writing – review and editing, D.M. and B.C.M.; supervision, B.C.M.; funding acquisition, B.C.M.

## Declaration of interests

The authors declare no competing interests.
